# Potential role of heat shock protein 90 (Hsp90) in HIV uncoating

**DOI:** 10.1186/1742-4690-8-S2-P80

**Published:** 2011-10-03

**Authors:** Pheroze Joshi, Cheryl A Stoddart

**Affiliations:** 1 Department of Medicine, University of California San Francisco, San Francisco, CA, 94110, USA

## Background

The selective pressure of antiretroviral therapy invariably results in the emergence of drug-resistant HIV variants that acquire mutations which can compromise the replication capacity of the virus. Recently, we showed that impaired replication of ritonavir-resistant HIV can be rescued by cellular activation. Ritonavir-resistance mutations in HIV protease prevent complete proteolysis of the Gag polypeptide, and the accumulation of uncleaved capsid-spacer peptide1 (CA-SP1) in maturing virus particles decreases the ability of HIV to replicate in human thymocytes and nonactivated cell lines. We confirmed that ritonavir-resistant HIV replication was arrested after virus entry but before complete proviral DNA synthesis. Using a functional genetic screen, we identified the abundant cellular chaperone Hsp90 as a host factor that can rescue the impaired replication of ritonavir-resistant HIV. We confirmed that Hsp90 expression increases in response to cellular activation and that pharmacologic inhibition of Hsp90 blocks HIV replication. Previous work has shown that altering key residues in structural domains of the HIV core prevents uncoating of the CA lattice in an infected cell. The CA mutations render the core either unstable or too stable, in each case resulting in abortive HIV replication at the uncoating stage of the virus life cycle. The nature of our genetic screen was based on the ability of a host factor to rescue a replication-impaired HIV isolate with a defective CA morphology. To verify Hsp90 as a potential host factor in HIV uncoating, we tested its ability to rescue the impaired replication of HIV with different destabilizing CA mutations.

## Materials and methods

Titrated virus stocks with mutations in different structural domains of HIV CA were generated. Jurkat T cells were transduced with WT and mutant Hsp90-expressing retrovirus and subsequently inoculated with the different CA mutants. Productive virus replication was monitored over 8 days.

## Results

Although the CA mutants replicated poorly in normal Jurkat T cells (0–27% relative to infectivity of WT HIV), they displayed varying degrees of impairment (32–75% relative to WT HIV) in PHA-activated human peripheral blood mononuclear cells and activated Jurkat T cells. Further, productive replication of several CA mutants were observed in Jurkat T cells transduced with WT Hsp90.

## Conclusions

The ability of CA-mutant HIV to replicate in Hsp90-transduced Jurkat T cells suggests a potential role for Hsp90 as an uncoating factor in postentry HIV replication. The increased expression of Hsp90 in activated T cells may override the destabilizing effects of CA mutations that arise in response to immune surveillance or drug therapy.

This project has been funded in part with Federal funds from NIAID, NIH, under Contract No. HHSN26620070002C/N01-AI-70002 and the California HIV/AIDS Research Program (CHRP) Grant No. ID09-SF-051 to C.A.S.

